# SMART-ESAS: Smartphone Monitoring and Assessment in Real Time of Edmonton Symptom Assessment System Scores for Patients With Cancer

**DOI:** 10.1200/GO.23.00447

**Published:** 2024-02-22

**Authors:** Chitrakshi Nagpal, Atul Sharma, Sameer Bakhshi, Prabhat Singh Malik, Hardik Gupta, Chetanya Mittal, Sneha Gund, Akash Kumar, Aparna Sharma, Deepam Pushpam, Sachin Khurana, Raja Pramanik, Nishkarsh Gupta, Atul Batra

**Affiliations:** ^1^ Department of Medical Oncology, Dr B.R.A. IRCH, All India Institute of Medical Sciences, New Delhi, India; ^2^ All India Institute of Medical Sciences, New Delhi, India; ^3^ Department of Medical Oncology, National Cancer Institute (NCI), All India Institute of Medical Sciences, Jhajjar, India; ^4^ Department of Onco-anaesthesia and Palliative Medicine, Dr B.R.A. IRCH, All India Institute of Medical Sciences, New Delhi, India

## Abstract

**PURPOSE:**

Serial patient-reported outcome (PRO) measurements in clinical practice are associated with a better quality of life and survival. Recording electronic PROs using smartphones is an efficient way to implement this. We aimed to assess the feasibility of the electronically filled Edmonton Symptom Assessment System (e-ESAS) scale in the lower-middle–income country (LMIC) setting.

**METHODS:**

Baseline clinical features and conventional paper-based ESAS (p-ESAS) were collected in newly diagnosed patients with solid organ tumors. Text message link was sent to these patients for filling e-ESAS. ESAS was categorized into physical, psychological, and total symptom domains. Scores were divided into none to mild (0-3) and moderate to severe (4-10). Intraclass correlation coefficients (ICCs) were used to determine the correlation between p-ESAS and e-ESAS. Multivariable logistic regression was used to identify independent factors affecting symptom burden.

**RESULTS:**

Of 1,160 participants who filled out p-ESAS, 595 completed both e-ESAS and p-ESAS questionnaires and were included in the final analysis. Moderate to severe physical, psychological, and total symptom scores were seen in 39.8%, 40%, and 39% of participants. Tiredness and anxiety were the most common physical and psychological symptoms, respectively. ICCs between the p-ESAS and e-ESAS varied between 0.75 and 0.9. Total symptom scores were independently predicted by metastatic disease (odds ratio [OR], 1.83; 95% CI, 1.26 to 2.67; *P* = .001) and a higher level of education (OR, 0.42; 95% CI, 0.25 to 0.72; *P* = .001).

**CONCLUSION:**

Paper-based and electronically filled ESASs have good intraobserver reliability across individual symptoms and domain scores in a representative cohort at a tertiary care institute in the LMIC. This may help us incorporate e-ESAS in routine clinical care in the real-world setting with financial, infrastructural, and manpower limitations.

## INTRODUCTION

Patients with cancer suffer from significant physical, psychological, and emotional symptoms that are attributed to both the disease and its treatment. Previous studies have demonstrated that 10-40% of patients have unrecognized distressing symptoms that hamper their quality of life.^1-4^ Patient-reported outcomes (PROs) are measurements of any aspect of a patient's health status that comes directly from the patient.^5^ They are a true reflection of the patient's physical, psychological, emotional, and social well-being, bypassing the interpretation of these responses by a health care provider, thus eliminating bias and interobserver variability.^6,7^

**CONTEXT**

**Key Objective**
Electronic patient-reported outcome (PRO) monitoring has been associated with improved quality of life and survival. Is it feasible to use electronic PROs in the lower-middle–income country (LMIC) setting?
**Knowledge Generated**
Smartphone-based PROs using the electronic Edmonton Symptom Assessment Scale (e-ESAS) were demonstrated to have a good correlation with the paper-based ESAS scores in patients with solid organ malignancies across all the domains. Maximum moderate to severe ESAS scores were seen in patients with lung and breast cancers, with metastatic disease and higher level of education being independently predictive of them.
**Relevance**
e-ESAS can be used in the LMIC setting to record, store, and easily access PROs in routine clinical practice. PRO monitoring may not be feasible because of financial, infrastructural, and manpower limitations, and e-ESAS is a pragmatic tool that might help us overcome these barriers.

The Edmonton Symptom Assessment System (ESAS)^8^ is one such scale designed for reporting, with intensity, the common symptoms of patients with cancer. It was originally developed in 1991 and has been revised and validated over the years for clinical and research purposes.^9^ It includes nine symptom domains, including six physical symptoms—pain, tiredness, nausea, drowsiness, appetite, and shortness of breath, and two psychological symptoms—depression and anxiety, along with one overall well-being. It has been validated in over 20 languages, including Hindi,^10-12^ and is widely used in oncologic settings, especially in advanced malignancies.^13^

PROs assist in documenting the symptom burden objectively, and their use in routine practice has been associated with better symptom control^4^ and quality of life,^14^ fewer hospitalizations, and better overall survival (OS).^6^ ESAS scores at baseline also have a prognostic value in metastatic cancers.^13^ Integration of these scales into clinical practice improves symptom control and helps tailor treatment for each patient.^4^ Since its inception, ESAS measurement has involved paper-based collection using visual analog scales and symptom assessment graphs.^8^ However, routine implementation of these scales necessitates an efficient and user-friendly way to collect data. Even with sufficient data to advocate the implementation of routine PRO monitoring in clinical practice, it might not be feasible in the outpatient setting in centers with very high patient load, especially in resource-limited settings.^15^

Electronic PRO (ePRO) reporting using web- or mobile-based surveys is a convenient method to collect symptom burden scores.^16^ Online self-reporting has proven to be an effective long-term strategy for monitoring symptoms and making treatment decisions as well.^17^ They have also been associated with significantly better completion rates,^18^ functional status,^19^ and overall survival^20,21^ compared with conventional surveillance using paper-based scales. Globally, more than 60% of users have shifted to using smartphones, including the lower-middle–income countries (LMICs) in the Asia-Pacific.^22^ According to the Global System for Mobile Communications Association's 2022 report, there are more than 1.5 billion smartphone users, with almost 90% having access to third generation (3G) or fourth generation (4G) network.^22,23^ Smartphone-based PROs can be a cost-effective way to monitor patients' symptoms, especially in resource-limited countries, where smartphone usage has increased significantly over the past few years. To our knowledge, there have been no data on the use of ePROs in LMICs, and their implementation and utility in such settings are largely unknown. We aimed to assess the feasibility and utility of the electronically filled ESAS scale (e-ESAS) in our setting. The objectives of the study were to (1) compare the consistency of PROs between the conventional paper-based ESAS questionnaire (p-ESAS) and e-ESAS filled on a smartphone and (2) analyze the clinical features associated with higher intensity of symptoms at baseline in patients with solid organ tumors.

## METHODS

### Study Design and Population

This was a prospective study conducted at a tertiary care center in India. All patients older than 18 years with newly diagnosed cancer registered at our cancer center, Dr B.R.A. IRCH, All India Institute of Medical Sciences, New Delhi, between July 2021 and July 2022 were screened. Our center caters to the Northern states of India, with a predominantly Hindi-speaking population. We register and treat approximately 10,000 new cancer cases annually. Among these, patients with a confirmed diagnosis of solid organ cancer, who were able to comprehend and answer the questionnaire as assessed by the study investigators on the basis of an initial conversation regarding educational status, were included. Patients with hematolymphoid malignancies or those who received previous treatment were excluded from the study. The study was approved by the Institute Ethics Committee before its conduct. An informed consent was obtained from all participants before inclusion in the study.

### Clinical Variables and ESAS Questionnaire

**FIG A1:**
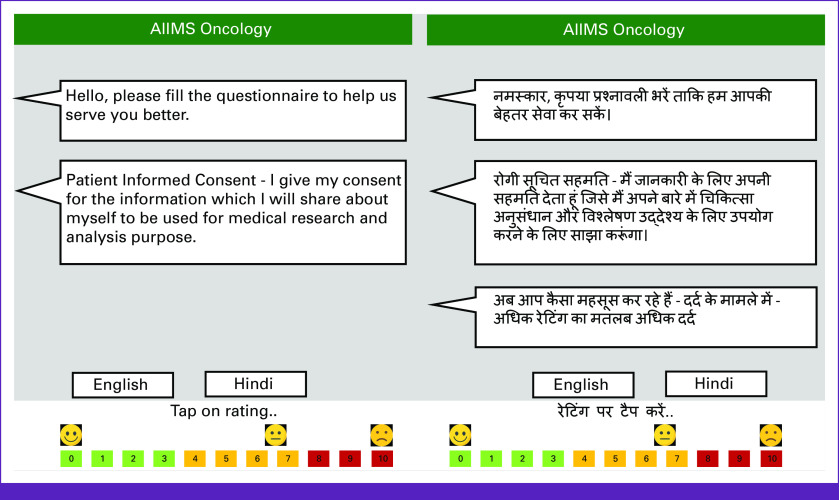
(Left) e-ESAS - format and visual analogue scale (VAS) used in English (Right) e-ESAS - format and VAS used in Hindi. AIIMS, All India Institute of Medical Sciences; e-ESAS, electronically filled Edmonton Symptom Assessment System.

Baseline demographics and clinical features, including age, sex, residence—rural or urban (as reported by the patients themselves), level of education, distance traveled to reach the hospital, underlying comorbid conditions, addictions, primary diagnosis, and stage of the disease, were recorded. Level of education was categorized into illiterate—someone who cannot read and write^24^; the ones who were literate were categorized into those who have completed primary, middle, or high school or those who have a graduation or postgraduation degree. Patients were asked to fill the ESAS in two formats—paper and electronic. All participants filled out the ESAS questionnaire on paper (p-ESAS) on the day of registration at our center. The e-ESAS questionnaire was then delivered as a text message with a unique link to those patients who possessed a smartphone (Appendix Fig A1). It could be answered either in English or in Hindi in both physical and digital formats on the basis of the patient’s language preference.^11^ Each patient was instructed to rate each symptom from zero to 10, with zero being no symptom at all and 10 representing the worst possible severity. If the patients were unable to complete it independently, however, they were capable of providing responses with assistance, then the caregiver or health care provider involved in the patient’s care was allowed to help.

Symptom scores were later segregated into two groups on the basis of severity: none to mild (0-3)^25^and moderate^4-6^ to severe.^7-10^ This was based on the receiver operating characteristic curve, which defined a cutoff of four for combined moderate to severe symptoms.^13,26,27^ They were also categorized into three groups—physical (first six domains), psychological (next two domains), and total symptom scores.^28,29^ Submitted p-ESAS questionnaires, which were <50% complete, were not included in the analysis. Incomplete questionnaires with more than 50% responses, subscores of the physical and psychological groups, and the total symptom scores were calculated using the average of the answered items.^13,27^ These were rounded off to the nearest integer and then divided into mild versus moderate to severe as above. For those that were completely filled, physical, psychological, and total scores were calculated by adding the individual domain scores and calculating the average scores. For e-ESAS, the software was developed such that submission was not allowed until all the questions had been answered; hence, complete responses were obtained for all patients.

### Statistics

Descriptive statistics were used to present demographics and baseline clinical details. Categorical data were described as percentages, and continuous variables were described as median with IQR. Comparison between groups was performed using the chi-square test for categorical variables and paired *t*-tests or the Wilcoxon signed-rank test for continuous data on the basis of the normality of the data distributed. The significant *P* value used was specified to be <.05 a priori.

Reliability assessment was performed using intraclass coefficients (ICCs, average measure, using the two-way mixed-effects model) between e-ESAS and p-ESAS to determine the correlation of the p-ESAS and e-ESAS measurements in each domain. Pooled mean and standard deviation were calculated, and ICC (or the tendency to be similar) was calculated as the ratio of the variance of interest to the total variance.^30^ By definition, ICC <0.5 is suggestive of poor reliability, 0.5-0.75 is moderate, 0.75-0.9 is good, and more than 0.9 is indicative of excellent reliability.^31^ Among the e-ESAS submissions, univariable logistic regression was applied to identify potential factors associated with moderate to severe symptoms. Multivariable analysis was performed on the factors in univariable analysis with *P* values ≤.1 to identify independent factors. Data were analyzed using StataCorp 2017 (Stata Statistical Software: Release 15, College Station, TX) and SPSS (IBM Corp: Released 2019, IBM SPSS Statistics for Windows, Version 26.0, Armonk, NY).

## RESULTS

### Patient Characteristics

During the study period between July 2021 and July 2022, a total of 1,160 eligible patients were screened for participation in the study and p-ESAS responses were collected. Of these patients, 634 patients completed the e-ESAS questionnaire sent on their phones. Five hundred twenty-six patients did not complete the e-ESAS questionnaire. This was because they did not possess a smartphone, or know how to use a smartphone even if they possessed one, or due to some technical glitch in receiving the text message despite it being sent multiple times. Few participants did not wish to fill the questionnaire one more time on their phones (after filling the physical one on paper).

**FIG 1:**
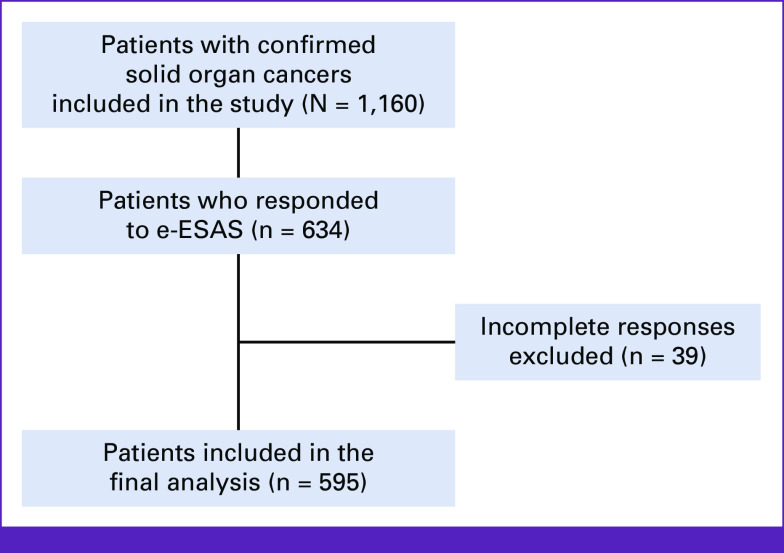
Workflow of the study. e-ESAS, electronically filled Edmonton Symptom Assessment System.

The dropout rates were not associated with any baseline factor, including rural residence, level of education, stage of cancer at baseline, or site of cancer. Thirty-nine patients had filled <50% of the p-ESAS forms, which were excluded from the analysis. Fifteen responses were incomplete, but contained more responses to more than 50% questions; therefore, these data were averaged and included in the analysis. A total of 595 patients were included in the final analysis (Fig 1).

The median age at diagnosis was 53 years (IQR, 44-63 years), and 307 (51.6%) patients were male. Twenty-five percent of patients were illiterate (n = 147), 23% resided in a rural area, and the median distance traveled by them to reach the hospital was 80 km (IQR, 25-300 km). The most common primary cancer was breast cancer (29.7%), followed in order of decreasing frequency by lung (29.2%), urothelial (13.3%), and GI (6.4%) cancers. More than 50% of patients presented with metastatic disease at baseline (Table 1).

**TABLE 1:** Baseline Characteristics

Baseline Characteristic	No. (%)
Age, years, median (IQR)	53 (44-63)
Sex	
Female	288 (48.4)
Male	307 (51.6)
Education level	
Illiterate	147 (24.7)
Primary/middle/high school	293 (49.2)
Graduate/postgraduate	148 (24.8)
Not available	7 (1.3)
Residence	
Urban	457 (76.8)
Rural	138 (23.2)
Distance from the health care center, km	
≤100	333 (56)
>100	262 (44)
Comorbidities^a^	
Hypertension	128 (21.5)
Diabetes mellitus	84 (14)
Hypothyroidism	23 (3.8)
Others^b^	60 (10)
None	370 (62.2)
Addictions	
Smoking	175 (29.4)
Alcohol use	90 (15)
Primary diagnosis	
Breast cancer	177 (29.7)
Lung cancer	174 (29.2)
Urothelial malignancies^c^	79 (13.3)
GI cancers^d^	38 (6.4)
Female genital tract^e^	31 (5.2)
Hepatobiliary carcinomas^f^	27 (4.5)
Others^g^	69 (11.6)
Stage at diagnosis	
Nonmetastatic	237 (39.8)
Stage I	19 (3.2)
Stage II	63 (10.6)
Stage III	155 (26)
Metastatic (stage IV)	321 (54)
Not available	37 (6.2)

Abbreviations: Ca, carcinoma; CAD, coronary artery disease; CVA, cerebrovascular accident; HCC, hepatocellular carcinoma; GB, gall bladder; RCC, renal cell carcinoma; SCC, squamous cell carcinoma.

^a^ No. may exceed 100% as some patients had more than one comorbidity.

^b^ Asthma, CVA, CAD.

^c^ Urinary bladder, prostate, RCC.

^d^ Esophagus, stomach, small intestine, colorectal, anal canal.

^e^ Ca of endometrium, ovary, cervix, and vagina.

^f^ Ca of GB, cholangiocarcinoma, Ca of pancreas, HCC.

^g^ Head and neck SCC, soft tissue sarcomas, osteosarcoma, carcinoid, Ca of penis.

### ESAS Responses and Correlation Between e-ESAS and p-ESAS

**FIG 2:**
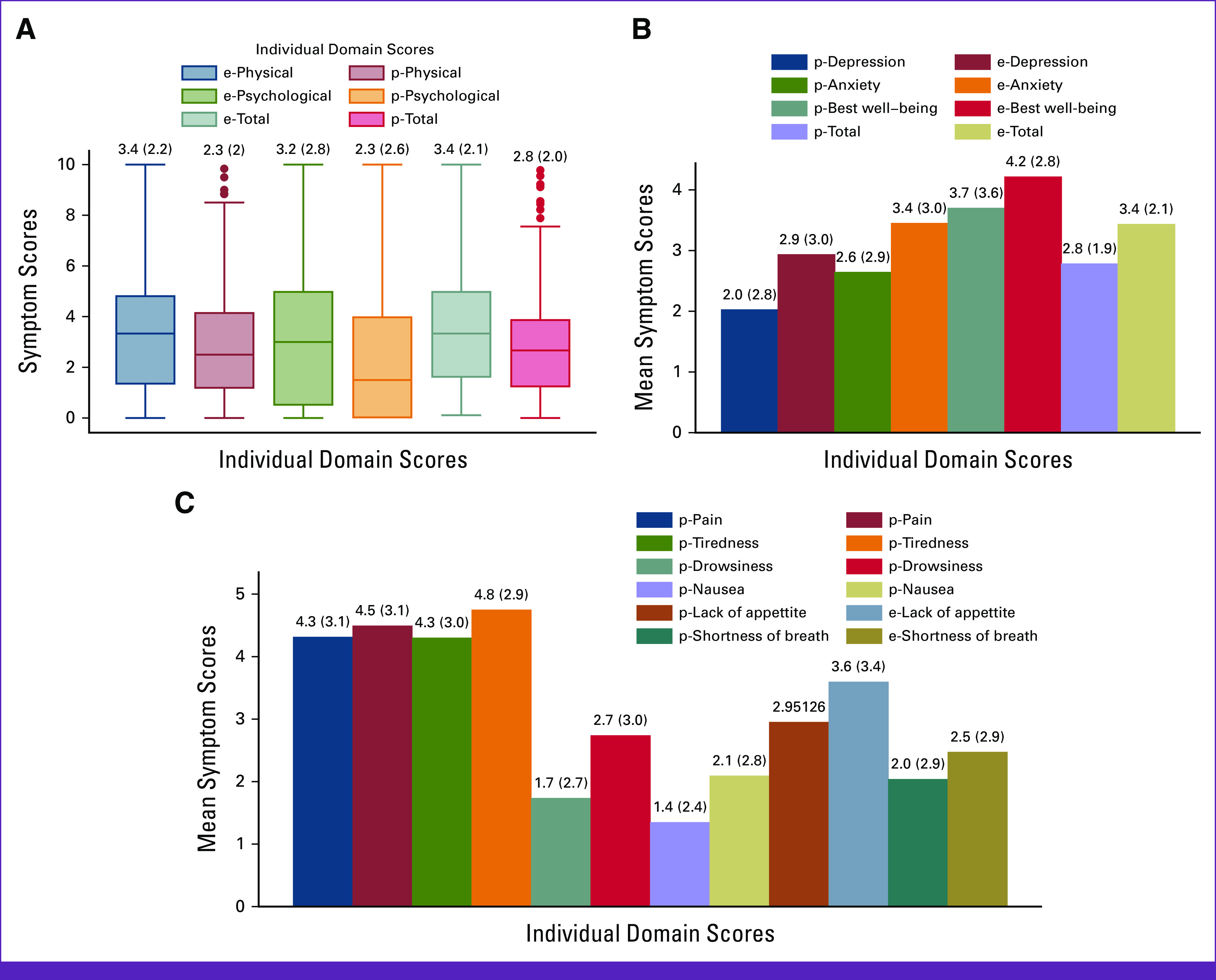
(A) Box plot showing physical, psychological, and overall symptom scores in e-ESAS and p-ESAS (mean ± SD labeled at the top of each box plot). (B) Bar graph showing means of individual psychological and best well-being domains in p-ESAS and e-ESAS (mean ± SD labeled at the top of each bar). (C) Bar graph showing means of individual physical domains in p-ESAS and e-ESAS (mean ± SD labeled at the top of each bar) e-ESAS, electronically filled Edmonton Symptom Assessment System; p-ESAS, paper-based Edmonton Symptom Assessment System; SD, standard deviation.

Individual category and domain-wise mean symptom scores are shown in Figures 2A-2C. Severity- and site-wise symptom scores are shown in Table 2. Moderate to severe physical symptoms were reported maximally by patients with lung cancer (49%, *P* < .001), followed by female genital tract (48.4%) and urothelial malignancies (43%) in decreasing frequency. Physical symptom intensity reported was similar for GI (37% moderate to severe), hepatobiliary (33.3%), and other malignancies. The most commonly reported physical symptom was tiredness, with 88% of patients experiencing the same. It was significantly more severe in those with metastatic disease. Moderate to severe psychological symptoms were numerically reported more in female genital tract cancers. However, there was no statistically significant difference in any other sites (*P* = .51). The most common psychological symptom reported was anxiety, seen in around 70% of all the patients, more commonly seen in those with stage IV disease.

**TABLE 2:** Smartphone-Based ESAS Severity Scores and for Different Primary Sites

(A) Smartphone-Based ESAS Severity Score		
Symptom	Severity: None to Mild, No. (%)	Severity: Moderate to Severe, No. (%)
Physical	358 (60.2)	237 (39.8)
Pain	241 (40.5)	354 (59.5)
Tiredness	217 (36.5)	378 (63.5)
Drowsiness	452 (76)	143 (24)
Nausea	493 (82.8)	102 (17.2)
Lack of appetite	359 (60.3)	236 (39.7)
Shortness of breath	430 (72.3)	165 (27.7)
Psychological	357 (60)	238 (40)
Depression	427 (71.8)	168 (28)
Anxiety	376 (63.2)	219 (36.8)
Best well-being	286 (48)	309 (52)
Other problems (n = 249)	140 (56.2)	109 (43.8)
Overall symptom score	363 (61)	232 (39)

(B) Smartphone-Based ESAS Severity Score for Different Primary Sites			
Primary Site	Physical	Psychological	Overall
	None-Mild, No. (%) Moderate-Severe, No. (%)	None-Mild, No. (%) Moderate-Severe, No. (%)	None-Mild, No. (%) Moderate-Severe, No. (%)
Breast (n = 177)	124 (70) 53 (30)	107 (59.8) 70 (41.2)	122 (68.1) 55 (31.9)
Lung (n = 174)	89 (51) 85 (49)	109 (62.6) 65 (37.4)	94 (54) 80 (46)
Urothelial (n = 79)	45 (57) 34 (43)	46 (58.2) 33 (41.8)	45 (57) 34 (43)
GI (n = 38)	24 (63) 14 (37)	21 (55.3) 17 (44.7)	25 (65.8) 13 (34.2)
Female genital tract (n = 31)	16 (51.6) 15 (48.4)	15 (48.4) 16 (51.6)	15 (48.4) 16 (51.6)
Hepatobiliary (n = 27)	18 (66.7) 9 (33.3)	20 (74) 7 (26)	21 (77.8) 6 (22.2)
Others	42 (60.2) 27 (39.8)	39 (56.5) 30 (43.5)	41 (59.4) 28 (40.6)

Abbreviation: ESAS, Edmonton Symptom Assessment System.

The total symptom scores were reported as moderate to severe in more than 50% patients with female genital tract cancers, followed by lung cancer. This was significantly higher than other sites (*P* = .025), which reported similar numbers of patients with moderate to severe symptoms. Site-wise individual symptom scores are shown in Appendix Table A1. For all patients, irrespective of the primary site of cancer, moderate to severe physical, psychological, and total symptom scores were seen in 39.8%, 40%, and 39% of the patients. The most common comments on other problems reported were constipation (n = 17 of 76, 22.4%) and gastritis (n = 15 of 76, 19.7%).

**FIG 3:**
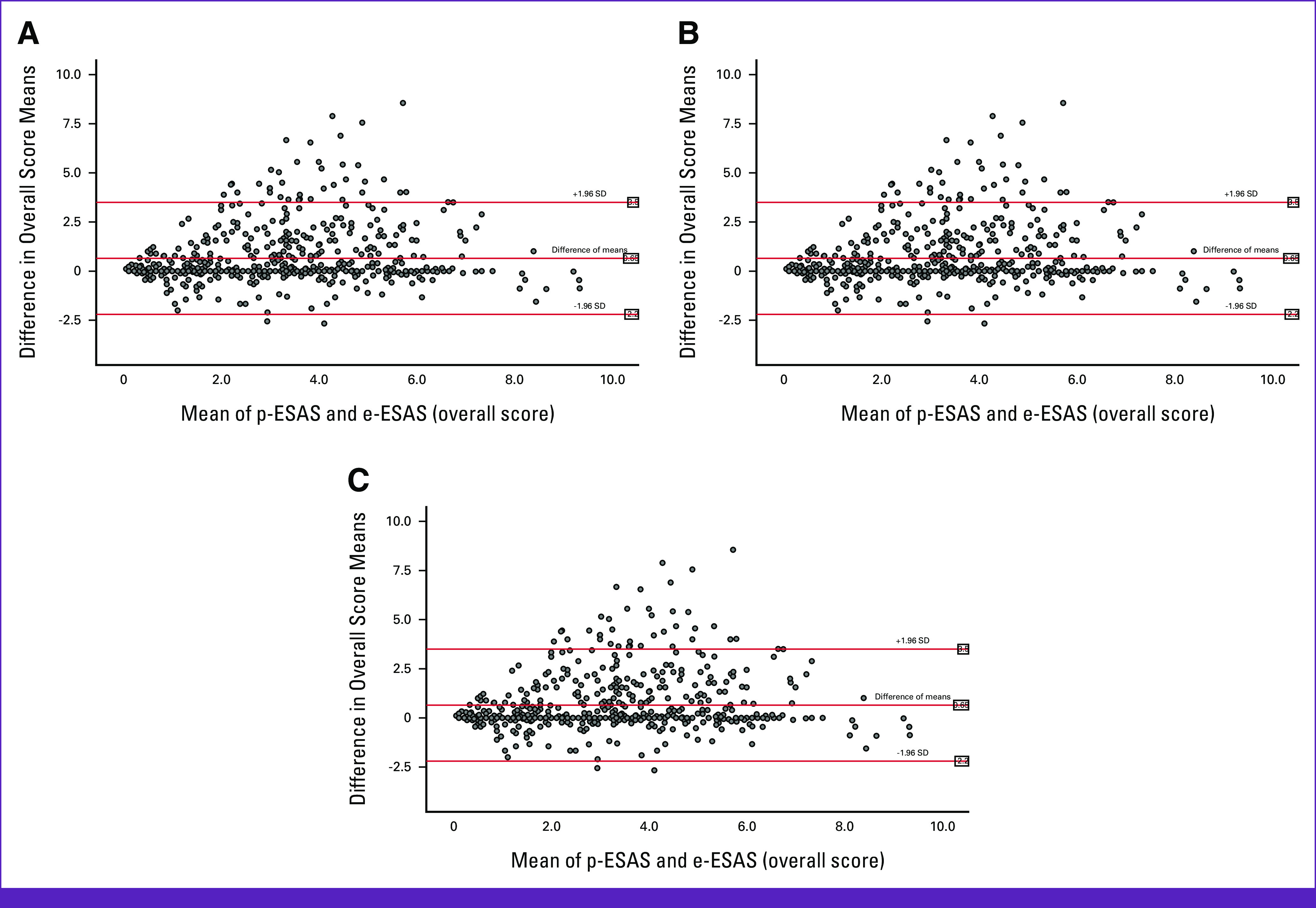
Bland-Altman plots for (A) total, (B) physical, and (C) psychological symptoms. e-ESAS, electronically filled Edmonton Symptom Assessment System; p-ESAS, paper-based Edmonton Symptom Assessment System; SD, standard deviation.

ICCs between the physically and online filled ESAS questionnaire (p-ESAS *v* e-ESAS) varied between 0.75 and 0.9 (Table 3). Bland-Altman plots to determine the difference between the two categories’ means for physical, psychological, and total symptom scores are shown in Figure 3. Graphically, most of the points fell between ±1.96 SD.

**TABLE 3:** Intraclass Correlation Coefficient Between p-ESAS and e-ESAS

Symptom	ICC (average measures)	95% CI	*P*	Mean (SD)	
				p-ESAS	e-ESAS
Physical	0.85	0.83 to 0.87	<.001	2.78 (2.00)	3.36 (2.18)
Pain	0.87	0.84 to 0.89	<.001	4.31 (3.08)	4.49 (3.09)
Tiredness	0.86	0.83 to 0.88	<.001	4.23 (2.97)	4.75 (2.91)
Drowsiness	0.74	0.69 to 0.78	<.001	1.73 (2.67)	2.74 (3.02)
Nausea	0.79	0.75 to 0.82	<.001	1.35 (2.42)	2.09 (2.84)
Lack of appetite	0.85	0.82 to 0.87	<.001	2.95 (3.42)	3.59 (3.41)
Shortness of breath	0.86	0.83 to 0.88	<.001	2.04 (2.83)	2.47 (2.90)
Psychological	0.84	0.82 to 0.87	<.001	2.33 (2.65)	3.19 (2.85)
Depression	0.83	0.80 to 0.86	<.001	2.02 (2.80)	2.93 (3.05)
Anxiety	0.82	0.77 to 0.83	<.001	2.64 (2.88)	3.45 (3.02)
Best well-being	0.81	0.77 to 0.84	<.001	3.70 (2.57)	4.21 (2.80)
Total symptom score	0.85	0.82 to 0.87	<.001	2.78 (1.91)	3.43 (2.14)

NOTE. Other symptoms category omitted in view of multiple mismatched observations.

Abbreviations: e-ESAS, electronically filled Edmonton Symptom Assessment System; ICC, intraclass correlation coefficient; p-ESAS, paper-based Edmonton Symptom Assessment System; SD, standard deviation.

### Factors Associated With Symptom Burden

On univariable analysis of the patients, rural residence; lung, urothelial, and hepatobiliary primary sites; education less than graduation level; and metastatic disease at baseline were associated with higher odds of moderate to severe total symptom scores. On multivariable analysis, only metastatic disease (odds ratio [OR], 1.92 [95% CI, 1.31 to 2.79]; *P* = .001) and higher level of education (OR, 0.42 [95% CI, 0.25 to 0.72]; *P* = .001) were significantly predictive of overall symptom burden.

For physical symptom scores, female sex; rural residence; smoking history; lung, urothelial, and hepatobiliary primary sites; education less than graduation level; and metastatic disease at baseline were associated with more moderate to severe physical symptom scores with univariable analysis. On multivariable analysis, only metastatic disease (OR, 1.83 [95% CI, 1.26 to 2.67]; *P* = .001) was significantly predictive of physical symptoms. Only higher level of education was predictive of lower moderate to severe psychological symptom scores (OR, 0.43 [95% CI, 0.26 to 0.69]; *P* = .001; Table 4).

**TABLE 4:** Multivariable Analysis for Association of Physical, Psychological, and Overall Symptom Scores With Baseline Characteristics

Factor	Total Score				Physical Scores				Psychological Scores			
	Univariable Analysis		Multivariable Analysis		Univariable Analysis		Multivariable Analysis		Univariable Analysis		Multivariable Analysis	
	OR (95% CI)	*P*	OR (95% CI)	*P*	OR (95% CI)	*P*	OR (95% CI)	*P*	OR (95% CI)	*P*	OR (95% CI)	*P*
Age (n = 595)	1.0 (0.99 to 1.01)	.28			1.00 (1.00 to 1.02)	.18			1.00 (0.98 to 1.01)	.75		
Sex (n = 595)	0.82 (0.59 to 1.15)	.26			0.68 (0.49 to 0.96)	**.03**	1.03 (0.60 to 1.77)	.92	1.22 (0.88 to 1.70)	.23		
Comorbidities	0.87 (0.62 to 1.22)	.41			0.84 (0.60 to 1.19)	.33			0.79 (0.56 to 1.10)	.17		
Residence												
Urban (n = 457)	Reference				Reference		Reference		Reference			
Rural (n = 138)	1.49 (1.01 to 2.19)	**.043**	1.16 (0.76 to 1.78)	.48	1.53 (1.04 to 2.25)	**.03**	1.24 (0.81 to 1.88)	.32	1.25 (0.85 to 1.84)	.25		
Education level												
Illiterate (n = 147)	Reference				Reference		Reference		Reference		Reference	
Schooling (n = 293)	0.8 (0.54 to 1.2)	.29	0.64 (0.42 to 1.00)	.052	0.91 (0.61 to 1.36)	**.037**	0.76 (0.49 to 1.18)	.226	0.67 (0.45 to 1.00)	**.053**	0.67 (0.45 to 1.00)	.053
Graduation (n = 148)	0.47 (0.29 to 0.77)	**.002**	0.42 (0.25 to 0.72)	**.001**	0.60 (0.38 to 0.97)	.118	0.59 (0.35 to 1.00)	.051	0.43 (0.26 to 0.69)	**.001**	0.43 (0.26 to 0.69)	**.001**
Primary site												
Breast (n = 179)	Reference		Reference		Reference		Reference		Reference			
Lung (n = 174)	1.89 (1.22 to 2.91)	**.004**	1.51 (0.94 to 2.43)	.09	2.23 (1.44 to 3.46)	**<.001**	1.61 (0.89 to 2.91)	.11	0.91 (0.59 to 1.40)	.67		
Urothelial (n = 79)	1.68 (0.97 to 2.10)	**.06**	1.36 (0.74 to 2.49)	.32	1.76 (1.02 to 3.06)	**.04**	1.26 (0.61 to 2.63)	.53	1.09 (0.64 to 1.88)	.74		
GI (n = 38)	1.15 (0.55 to 2.42)	.71	1.00 (0.44 to 2.23)	.99	1.36 (0.65 to 2.84)	.41	1.03 (0.44 to 2.43)	.95	1.24 (0.61 to 2.51)	.56		
Female genital (n = 31)	0.63 (0.24 to 1.66)	.35	0.44 (0.15 to 1.25)	.12	1.17 (0.49 to 2.77)	.72	0.86 (0.34 to 2.20)	.76	0.54 (0.21 to 1.33)	.18		
Hepatobiliary (n = 27)	2.37 (1.09 to 5.12)	**.03**	2.16 (0.95 to 4.92)	.07	2.19 (1.01 to 4.76)	**.047**	1.89 (0.83 to 4.31)	.13	1.63 (0.76 to 3.51)	.21		
Others (n = 69)	1.51 (0.85 to 2.70)	.16	1.4 (0.76 to 2.60)	.28	1.50 (0.84 to 2.69)	.168	1.23 (0.62 to 2.46)	.55	1.18 (0.67 to 2.07)	.57		
Smoking history (n = 175)	1.30 (0.91 to 1.86)	.15			1.61 (1.13 to 2.30)	**.01**	1.36 (0.85 to 2.19)	.20				
Alcohol intake (n = 90)	1.05 (0.66 to 1.66)	.83			1.32 (0.84 to 2.07)	.23			0.71 (0.44 to 1.14)	.16		
Metastatic disease *v* localised	1.99 (1.39 to 2.84)	**<.001**	1.91 (1.31 to 2.79)	**.001**	2.01 (1.41 to 2.87)	**<.001**	1.83 (1.26 to 2.67)	**.001**	1.31 (0.93 to 1.86)	.12		

NOTE. Factors with significant *P* values (<.1 for univariable analysis and <.05 for multivariable analysis) are in bold.

Abbreviation: OR, odds ratio.

## DISCUSSION

Our study aimed to evaluate the feasibility and utility of using a mobile-based secure system for monitoring ePROs. Despite their vast clinical utility,^20,21,32^ the assessment of PROs is not prevalent, given the logistic strain it places on health care delivery systems. In this regard, electronic reporting is a pragmatic solution with additional benefits as well. Randomized controlled trials have proven the superiority of ePROs in the developed nations^19,21^; however, to our knowledge, there are no supporting real-world data on PROs collected electronically from LMICs like India, where high cancer burden and resource-limited setting make ePROs a necessity. ESAS questionnaire, developed over 30 years back, has been widely used and validated in oncologic and palliative settings.^8,12,33^ We found a good reliability (ICC varying between 0.74 and 0.87, *P* < .001) between e-ESAS and p-ESAS across individual symptoms and domain scores obtained in our population.

We also found that the electronically filled ESAS scores were consistently higher than the physical ones. This might be because the e-ESAS scores were filled after the physical questionnaire, and by that time, the participants would have received medications to palliate symptoms, which might have improved the symptoms compared with the first contact.

Electronic systems allow easy compilation and storage of patient symptom scores, easier standardization, and longitudinal follow-up.^16^ Therefore, electronic PROs (e-ESAS) may be incorporated in routine clinical practice, with the additional benefit of avoiding a dedicated clinic visit for PRO measurements, which is especially important for high-burden centers.

Implementation of eESAS, however, might be encountered with some difficulties. Nearly half of the participants in our study did not complete the online form, despite 95% of them possessing smartphones. Regression analysis did not reveal any association with the level of education, urban or rural residence, or metastatic/localized disease. The speculated reasons include an inability to comprehend the questions or the lack of patient motivation, which may be due to a higher symptom burden, a sense of hopelessness, or poor social support and not possessing a smartphone in a minority of patients. These shortcomings, however, may be overcome with the help of health care providers,^34^ who can periodically encourage patients and train them to ensure high completion rates. This might also improve with a better understanding of the e-ESAS tool with future research.

Among the participants who filled the e-ESAS scale, moderate to severe physical and overall symptoms were reported maximally by patients with lung and female genital tract cancers. Similar results have been seen in multiple studies validating paper-based ESAS,^13,27,35^ as are seen with our e-ESAS. We also found that metastatic disease at presentation predicted a higher symptom burden, as shown in previous studies as well.^27,36^ Higher education was independently predictive of lower symptom burden. This is in contrast to previous data;^27^ however, our results may be biased by the fact that more educated patients filled the e-ESAS compared with the illiterate ones.

The strengths of our study include its prospective nature, the representativeness of the enrolled cohort, and the documentation of real-time data collection. To the best of our knowledge, it is the first study that has tried to validate the use of ePROs in the LMIC setting. It was, however, limited by the number of smartphone-based responses—only 634 patients of 1,160 responded to the e-ESAS link sent via text message. This might question the generalizability of the study. In addition, there was no formal assessment of the patients’ cognitive abilities before recording their responses, only a subjective idea of which participant would be able to comprehend the questions. In addition, it was a one-time assessment of the PROs, predominantly for validation of the electronic measurements, and there was no longitudinal follow-up. Another limitation of the study is the presence of baseline metastasis in more than half of the total number of patients, which is slightly higher than the Indian epidemiologic data.^37^ This can be explained by the presence of a referral bias in the outpatient department of a tertiary care center as ours. The ESAS scale itself also has some inherent limitations,^33^ such as the difficulty in differentiating between drowsiness and tiredness; reversal of scale for lack of appetite where zero might be coded as the worst and 10 as the best, as opposed to the actual scale; and general understanding of categories like best well-being.

A majority of patients with solid organ cancers present with up-front metastatic disease,^37^ where the intent of treatment is palliative, and primary goals of care include symptom control and improvement in the quality of life. Ironically, for these patients, periodic visits to the out-patient department for PRO monitoring are more difficult since they have higher symptom scores to begin with. Apart from this, serial symptom measurements (in palliative and curative settings) have shown to improve quality of life and OS. PRO monitoring may not be feasible because of financial, infrastructural, and manpower limitations in our settings. e-ESAS is one such pragmatic tool to help us overcome these barriers. With encouraging results from our study, we may extend the use of e-ESAS in routine clinical care to help us tailor therapy for each patient and improve outcomes beyond the traditional biochemical or radiologic indices. However, evaluation of the cause of high dropout rates for e-ESAS via interviews and thematic analysis represents the next step in expanding the reach of this pragmatic assessment method.

## Data Availability

Anonymized patient-level data that support the findings of the study will be available from the corresponding author on reasonable request.

## APPENDIX

**TABLE A1:** Individual Symptom Scores According to Primary Site

Primary Site (No.)	Pain	Tiredness	Drowsiness	Nausea	LOA	SOB	Depression	Anxiety
	None-Mild, No. (%) Moderate-Severe, No. (%)	None-Mild, No. (%) Moderate-Severe, No. (%)	None-Mild, No. (%) Moderate-Severe, No. (%)	None-Mild, No. (%) Moderate-Severe, No. (%)	None-Mild, No. (%) Moderate-Severe, No. (%)	None-Mild, No. (%) Moderate-Severe, No. (%)	None-Mild, No. (%) Moderate-Severe, No. (%)	None-Mild, No. (%) Moderate-Severe, No. (%)
Breast (n = 177)	87 (49) 90 (51)	84 (47.4) 93 (52.6)	148 (83.6) 29 (17.4)	158 (89.2) 19 (10.8)	139 (78.5) 38 (21.5)	144 (81.4) 33 (19.6)	125 (70.6) 52 (29.4)	114 (64.4) 63 (35.6)
Lung (n = 174)	55 (31.6) 119 (68.4)	40 (23) 134 (77)	135 (77.6) 39 (22.4)	145 (83.3) 29 (16.7)	82 (47) 92 (53)	84 (48.3) 90 (51.7)	130 (74.7) 44 (25.3)	111 (63.8) 63 (36.2)
Urothelial (n = 79)	33 (41.8) 46 (58.2)	30 (38) 49 (62)	56 (70.9) 23 (29.1)	62 (78.5) 17 (21.5)	48 (60) 31 (40)	66 (83.5) 13 (16.5)	58 (73.4) 21 (26.6)	54 (68.4) 25 (31.6)
GI (n = 38)	17 (44.7) 21 (56.3)	17 (44.7) 21 (56.3)	26 (68.4) 12 (31.6)	29 (76.3) 9 (23.7)	18 (47.3) 20 (52.7)	37 (97.3) 1 (2.7)	27 (71) 11 (29)	26 (68.4) 12 (31.6)
Female genital tract (n = 31)	10 (32.2) 21 (67.8)	13 (42) 18 (58)	19 (61.3) 12 (38.7)	21 (67.8) 10 (32.2)	19 (61.3) 12 (38.7)	24 (77.4) 7 (22.6)	20 (64.5) 11 (35.5)	17 (54.8) 14 (45.2)
Hepatobiliary (n = 27)	13 (48) 14 (52)	10 (37) 17 (63)	20 (74) 7 (26)	21 (77.8) 6 (22.3)	13 (48) 14 (52)	22 (81.5) 5 (18.5)	20 (74) 7 (26)	16 (59.2) 11 (40.8)

Abbreviations: LOA, loss of appetite; SOB, shortness of breath.

## References

[1] Carlson LE, Angen M, Cullum J, et al: High levels of untreated distress and fatigue in cancer patients. Br J Cancer 90:2297-2304, 2004 15162149 10.1038/sj.bjc.6601887 PMC2410292

[2] Laugsand EA, Sprangers MAG, Bjordal K, et al: Health care providers underestimate symptom intensities of cancer patients: A multicenter European study. Health Qual Life Outcomes 8:104, 2010 20858248 10.1186/1477-7525-8-104 PMC2949821

[3] Sanson-Fisher R, Girgis A, Boyes A, et al: The unmet supportive care needs of patients with cancer. Supportive Care Review Group. Cancer 88:226-237, 2000 10618627 10.1002/(sici)1097-0142(20000101)88:1<226::aid-cncr30>3.3.co;2-g

[4] Howell D, Molloy S, Wilkinson K, et al: Patient-reported outcomes in routine cancer clinical practice: A scoping review of use, impact on health outcomes, and implementation factors. Ann Oncol 26:1846-1858, 2015 25888610 10.1093/annonc/mdv181

[5] US Department of Health and Human Services FDA Center for Drug Evaluation and Research; US Department of Health and Human Services FDA Center for Biologics Evaluation and Research; US Department of Health and Human Services FDA Center for Devices and Radiological Health: Guidance for industry: Patient-reported outcome measures: Use in medical product development to support labeling claims: Draft guidance. Health Qual Life Outcomes 4:79, 2006 17034633 10.1186/1477-7525-4-79 PMC1629006

[6] Kerrigan K, Patel SB, Haaland B, et al: Prognostic significance of patient-reported outcomes in cancer. JCO Oncol Pract 16:e313-e323, 2020 32048943 10.1200/JOP.19.00329 PMC7846047

[7] Sørensen JB, Klee M, Palshof T, et al: Performance status assessment in cancer patients. An inter-observer variability study. Br J Cancer 67:773-775, 1993 8471434 10.1038/bjc.1993.140 PMC1968363

[8] Bruera E, Kuehn N, Miller MJ, et al: The Edmonton Symptom Assessment System (ESAS): A simple method for the assessment of palliative care patients. J Palliat Care 7:6-9, 1991 1714502

[9] Watanabe SM, Nekolaichuk C, Beaumont C, et al: A multicenter study comparing two numerical versions of the Edmonton Symptom Assessment System in palliative care patients. J Pain Symptom Manage 41:456-468, 2011 20832987 10.1016/j.jpainsymman.2010.04.020

[10] Hui D, Shamieh O, Paiva CE, et al: Minimal clinically important differences in the Edmonton Symptom Assessment Scale in cancer patients: A prospective, multicenter study. Cancer 121:3027-3035, 2015 26059846 10.1002/cncr.29437 PMC4595042

[11] Yogananda MN, Muthu V, Prasad KT, et al: Utility of the revised Edmonton Symptom Assessment System (ESAS-r) and the Patient-Reported Functional Status (PRFS) in lung cancer patients. Support Care Cancer 26:767-775, 2018 29027005 10.1007/s00520-017-3887-1

[12] Hui D, Bruera E: The Edmonton Symptom Assessment System 25 years later: Past, present and future developments. J Pain Symptom Manage 53:630-643, 2017 28042071 10.1016/j.jpainsymman.2016.10.370 PMC5337174

[13] Batra A, Yang L, Boyne DJ, et al: Associations between baseline symptom burden as assessed by patient-reported outcomes and overall survival of patients with metastatic cancer. Support Care Cancer 29:1423-1431, 2021 32676854 10.1007/s00520-020-05623-6

[14] Clarijs ME, Thurell J, Kühn F, et al: Measuring quality of life using patient-reported outcomes in real-world metastatic breast cancer patients: The need for a standardized approach. Cancers 13:2308, 2021 34065805 10.3390/cancers13102308 PMC8151772

[15] International Agency for Research on Center: Cancer Today. https://gco.iarc.fr/today/online-analysis-multi-bars?mode=cancer&mode_population=continents&population=356&sex=0&cancer=29&type=0&statistic=0&prevalence=0&color_patette=default

[16] Schwartzberg L: Electronic patient-reported outcomes: The time is ripe for integration into patient care and clinical research. Am Soc Clin Oncol Educ Book 35:e89-e96, 2016 27249775 10.1200/EDBK_158749

[17] Basch E, Iasonos A, Barz A, et al: Long-term toxicity monitoring via electronic patient-reported outcomes in patients receiving chemotherapy. J Clin Oncol 25:5374-5380, 2007 18048818 10.1200/JCO.2007.11.2243

[18] Basch E, Stover AM, Schrag D, et al: Clinical utility and user perceptions of a digital system for electronic patient-reported symptom monitoring during routine cancer care: Findings from the PRO-TECT trial. JCO Clin Cancer Inform 10.1200/CCI.20.00081 10.1200/CCI.20.00081 PMC7768331 33112661

[19] Basch E, Schrag D, Henson S, et al: Effect of electronic symptom monitoring on patient-reported outcomes among patients with metastatic cancer: A randomized clinical trial. JAMA 327:2413-2422, 2022 35661856 10.1001/jama.2022.9265 PMC9168923

[20] Denis F, Basch E, Septans AL, et al: Two-year survival comparing web-based symptom monitoring vs routine surveillance following treatment for lung cancer. JAMA 321:306-307, 2019 30667494 10.1001/jama.2018.18085 PMC6439676

[21] Basch E, Deal AM, Dueck AC, et al: Overall survival results of a trial assessing patient-reported outcomes for symptom monitoring during routine cancer treatment. JAMA 318:197-198, 2017 28586821 10.1001/jama.2017.7156 PMC5817466

[22] McCool J, Dobson R, Whittaker R, et al: Mobile health (mHealth) in low- and middle-income countries. Annu Rev Public Health 43:525-539, 2022 34648368 10.1146/annurev-publhealth-052620-093850

[23] GSMA: The Mobile Economy Asia Pacific 2023. The Mobile Economy. https://www.gsma.com/mobileeconomy/asiapacific/

[24] India 2020: Reference Annual. Profile—Literacy—Know India: National Portal of India. Publications Division, Ministry of Information and Broadcasting, Government of India, 2020. https://knowindia.india.gov.in/profile/literacy.php

[25] Hui D, Park M, Shamieh O, et al: Personalized symptom goals and response in patients with advanced cancer. Cancer 122:1774-1781, 2016 26970366 10.1002/cncr.29970 PMC4873446

[26] Selby D, Cascella A, Gardiner K, et al: A single set of numerical cutpoints to define moderate and severe symptoms for the Edmonton Symptom Assessment System. J Pain Symptom Manage 39:241-249, 2010 19963335 10.1016/j.jpainsymman.2009.06.010

[27] Cuthbert CA, Boyne DJ, Yuan X, et al: Patient-reported symptom burden and supportive care needs at cancer diagnosis: A retrospective cohort study. Support Care Cancer 28:5889-5899, 2020 32270311 10.1007/s00520-020-05415-y

[28] Hui D, Shamieh O, Paiva CE, et al: Minimal clinically important difference in the physical, emotional, and total symptom distress scores of the Edmonton Symptom Assessment System. J Pain Symptom Manage 51:262-269, 2016 26482223 10.1016/j.jpainsymman.2015.10.004 PMC4733575

[29] Cheung WY, Le LW, Zimmermann C: Symptom clusters in patients with advanced cancers. Support Care Cancer 17:1223-1230, 2009 19184126 10.1007/s00520-009-0577-7

[30] Liljequist D, Elfving B, Skavberg Roaldsen K: Intraclass correlation—A discussion and demonstration of basic features. PLoS ONE 14:e0219854, 2019 31329615 10.1371/journal.pone.0219854 PMC6645485

[31] Koo TK, Li MY: A guideline of selecting and reporting intraclass correlation coefficients for reliability research. J Chiropr Med 15:155-163, 2016 27330520 10.1016/j.jcm.2016.02.012 PMC4913118

[32] Hellewell J, Abbott S, Gimma A, et al: Feasibility of controlling COVID-19 outbreaks by isolation of cases and contacts. Lancet Glob Health 8:e488-e496, 2020 32119825 10.1016/S2214-109X(20)30074-7 PMC7097845

[33] Watanabe S, Nekolaichuk C, Beaumont C, et al: The Edmonton Symptom Assessment System—What do patients think? Support Care Cancer 17:675-683, 2009 18953577 10.1007/s00520-008-0522-1

[34] Basch E, Deal AM, Kris MG, et al: Symptom monitoring with patient-reported outcomes during routine cancer treatment: A randomized controlled trial. J Clin Oncol 34:557-565, 2016 26644527 10.1200/JCO.2015.63.0830 PMC4872028

[35] Cheung WY, Le LW, Gagliese L, et al: Age and gender differences in symptom intensity and symptom clusters among patients with metastatic cancer. Support Care Cancer 19:417-423, 2011 20333411 10.1007/s00520-010-0865-2

[36] Jensen RE, Potosky AL, Moinpour CM, et al: United States population-based estimates of patient-reported outcomes measurement information system symptom and functional status reference values for individuals with cancer. J Clin Oncol 35:1913-1920, 2017 28426375 10.1200/JCO.2016.71.4410 PMC5466008

[37] Mathur P, Sathishkumar K, Chaturvedi M, et al: Cancer statistics, 2020: Report from national cancer registry programme, India. JCO Glob Oncol 10.1200/GO.20.00122 10.1200/GO.20.00122 PMC7392737 32673076

